# Outcomes of COVID-19 in the Omicron-predominant wave: large-scale real-world data analysis with a comparison to influenza

**DOI:** 10.1186/s41479-025-00158-y

**Published:** 2025-02-05

**Authors:** Koichi Miyashita, Hironao Hozumi, Kazuki Furuhashi, Eiji Nakatani, Yusuke Inoue, Hideki Yasui, Yuzo Suzuki, Masato Karayama, Noriyuki Enomoto, Tomoyuki Fujisawa, Naoki Inui, Toshiyuki Ojima, Takafumi Suda

**Affiliations:** 1 Second Division, Department of Internal Medicine, Hamamatsu University School of Medicine, 1-20-1 Handayama, Chuo-ku, Hamamatsu, 431-3192 Japan; 2 Department of Biostatistics and Data Science, Graduate School of Medical Science, Nagoya City University, 1 Kawasumi, Mizuho-cho, Mizuho-ku, Nagoya, 467-8601 Japan; 3 Department of Clinical Pharmacology and Therapeutics, Hamamatsu University School of Medicine, 1-20-1 Handayama, Chuo-ku, Hamamatsu, 431-3192 Japan; 4 Department of Community Health and Preventive Medicine, Hamamatsu University School of Medicine, 1-20-1 Handayama, Chuo-ku, Hamamatsu, 431-3192 Japan

**Keywords:** COVID-19, Influenza, Mortality, National Database of Health Insurance Claims and Specific Health Checkups of Japan, NDB

## Abstract

**Purpose:**

Studies on COVID-19 mortality during the Omicron-predominant wave have focused primarily on the inpatient/emergency room setting, and real-world data including both inpatients and outpatients are lacking.

**Methods:**

Patients diagnosed with COVID-19 (*n* = 27,440,148) or influenza (*n* = 8,179,641) from January 2020 to April 2023 were identified using nationwide claims data in Japan. Patients with COVID-19 in the Omicron-predominant wave were compared with their counterparts in earlier waves, and a subset of the former group (May 2022–April 2023) was compared with patients with influenza as controls.

**Results:**

The mortality rates (average number of deaths/cases per week) of COVID-19 decreased over time, being 2.7% (169/6312), 2.1% (397/18,754), 0.7% (195/28,273), and 0.4% (1613/378,848) in the wild-type–, Alpha-, Delta-, and Omicron-predominant waves, respectively. However, the number of deaths increased substantially in the Omicron-predominant wave, especially among the elderly (e.g., in the Delta- and Omicron-predominant waves, the average numbers of deaths/cases per week were < 1/5527 (< 0.01%) and 4/105,763 (< 0.01%) respectively, in patients aged 0–19, versus 101/925 (10.9%) and 1212/20,771 (5.8%), respectively, in patients aged ≥ 80). The mortality rate was lower for patients with COVID-19 than in those with influenza among those aged ≤ 39 years but higher among those aged ≥ 40 years.

**Conclusions:**

In the Omicron-predominant wave, the mortality rate of COVID-19 decreased, but the number of patients increased, leading to a substantial increase in the number of deaths, especially among the elderly. The mortality rate of COVID-19 was higher than that of influenza in the elderly but not in the young, highlighting the need for age-specific interventions.

**Supplementary Information:**

The online version contains supplementary material available at 10.1186/s41479-025-00158-y.

## Introduction

Coronavirus disease 2019 (COVID-19), caused by severe acute respiratory syndrome coronavirus 2 (SARS-CoV-2), rapidly spread globally, and a pandemic was declared by the World Health Organization (WHO) in March 2020 [1]. Subsequently, SARS-CoV-2 mutated, and several variants were designated as variants of concern (VOCs) [2]. The infections caused by these variants led to a significant disease burden internationally, resulting in huge numbers of deaths globally. The Omicron variant was first reported in November 2021, and it was designated the fifth VOC. By this time, vaccines against COVID-19 were widely available, treatments had improved, and this variant proved less virulent than the earlier prevalent variants [3–6]. Thus, the WHO ended its declaration of a pandemic in May 2023 [1]. However, the COVID-19 Omicron epidemic continues, remaining a major societal problem. Further understanding of the COVID-19 Omicron variant is needed to take measures against this disease.

Since the early pandemic period, studies have been conducted using influenza as a control disease to clarify the clinical characteristics and outcomes of patients with COVID-19 [7–15]. However, these studies were limited to hospitalized patients or those who visited the emergency department. Given that most patients with COVID-19 or influenza are treated in outpatient settings at clinics or family physicians’ offices, a real-world study with large-scale data covering the entire patient population is essential to more accurately understand the current status of these infectious diseases. The National Database of Health Insurance Claims and Specific Health Checkups of Japan (NDB) is a nationwide medical database that contains almost all claim data for people residing in Japan. To determine the characteristics and outcomes of patients with COVID-19 in the Omicron-predominant wave, we compared them to those infected in earlier waves and to patients with influenza in the Omicron-predominant wave using the NDB.

## Methods

### Dataset and patients

The NDB covers > 99% of Japanese claims data, including both inpatient and outpatient claims [16]. Given the fact that almost all people in Japan are covered by insurance, data on nearly all patients diagnosed with COVID-19 or influenza can be extracted from this database. Thus, the use of the NDB permits studies with external validity that represent the real world. The NDB contains data on patients’ age, sex, diseases based on the International Statistical Classification of Diseases and Related Health Problems, 10th revision (ICD-10), medical procedures covered by insurance, and mortality. However, it does not contain information on smoking history, vaccinations for COVID-19 or influenza, laboratory/physiological findings, and causes of death. In this study, we extracted anonymized data on patients diagnosed with COVID-19 (ICD-10 code U07) or influenza (ICD-10 code J09–J11) from 1 January 2020 to 30 June 2023 from the NDB. We also extracted data on diseases listed in the Charlson comorbidity index (Supplementary Table 1) that were previously diagnosed (i.e., recorded in the database) before the diagnosis of COVID-19 or influenza and still present at the time of COVID-19 or influenza diagnosis for each patient. This index has been widely used for evaluating risk adjustment in outcome studies [17]. Information on oxygen supplementation, high-flow nasal canula (HFNC) therapy, mechanical ventilation, and extracorporeal membrane oxygenation use within 10 days of the index date, defined as the date of a COVID-19 or influenza diagnosis, was extracted to assess respiratory supportive care. Death was defined as all-cause death within 60 days of a COVID-19 or influenza diagnosis.

### Waves

The NDB does not include information on the SARS-CoV-2 variants confirmed in each patient. As in our previous reports [18, 19], based on the survey of the variants detected in Tokyo, Japan, any VOC detected in more than 50% of the performed tests was defined as the predominant VOC [20]. The waves of the study period were as follows: wild-type–predominant, 1 January 2020–18 April 2021; Alpha-predominant, 19 April 2021–18 July 2021; Delta-predominant, 19 July 2021–3 January 2022; and Omicron-predominant, 4 January 2022–30 April 2023.

### Statistical analysis

Categorical variables are expressed as numbers (%). To compare mortality rates between two groups, the risk ratios and corresponding 95% confidence intervals (CIs) were calculated using Poisson regression models. Unadjusted risk ratios were also calculated for each age group, and adjusted risk ratios were calculated using multivariable Poisson regression models adjusted for sex and comorbidities. *P* < 0.05 was considered statistically significant. However, because of the large sample size in this study, absolute standardized differences (ASDs) were presented to assess differences in the baseline characteristic variables between two groups. When ASD < 0.1, the variables between the two groups were taken as approximately equivalent even if the *P*-value was significant. All data were analyzed using R version 4.3.0 (R Core team, Vienna, Austria).

## Results

### Weekly number of cases and deaths among patients with COVID-19 and influenza

**Fig. 1:**
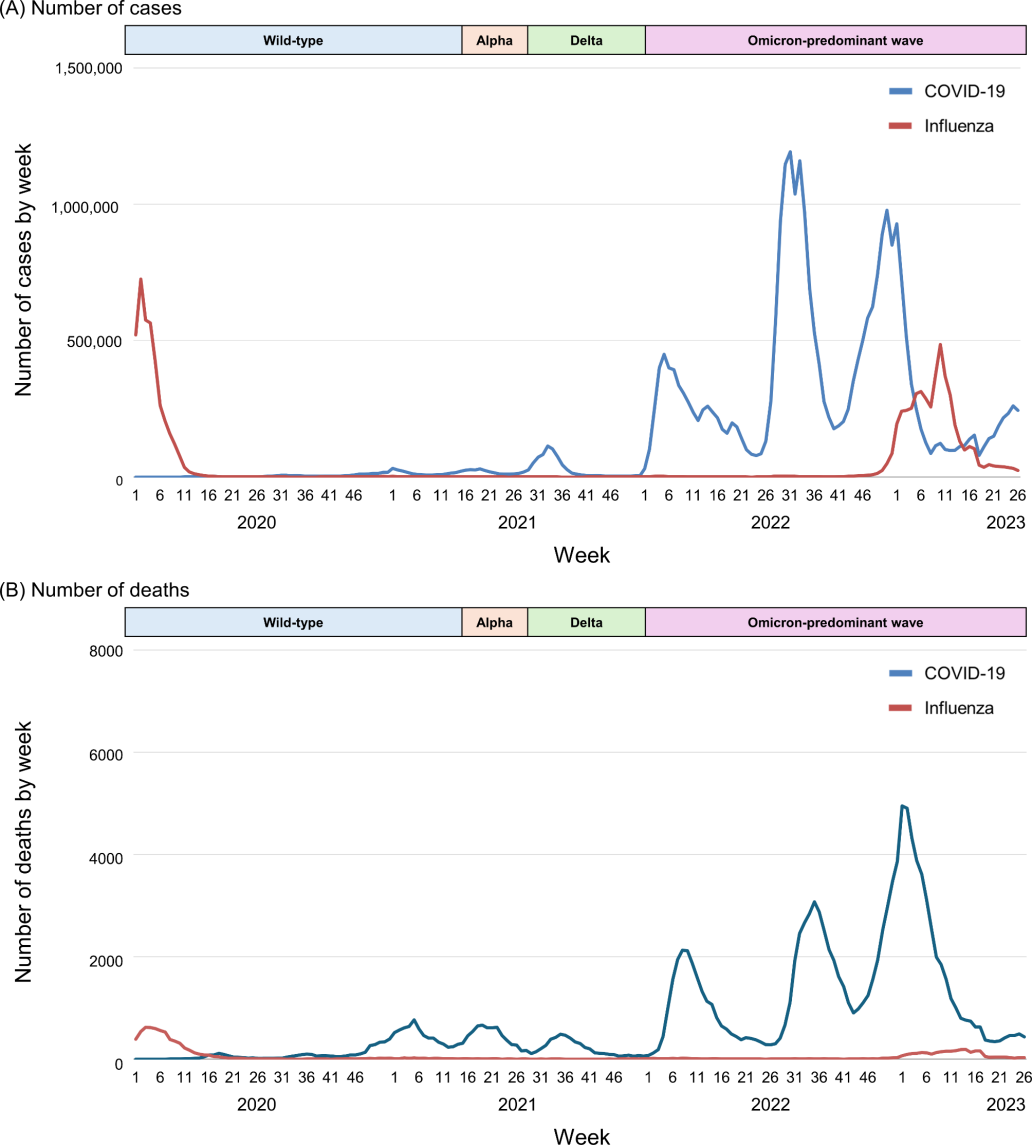
Weekly number of cases and deaths of COVID-19 and influenza from the early pandemic to June 2023. (**A**) The weekly number of cases of COVID-19 and influenza from the early pandemic to June 2023. During the Omicron-predominant wave, the maximum weekly number of patients with COVID-19 was 1,192,169, occurring in the 31st week of 2022, whereas the maximum weekly number of patients with influenza was 486,109, occurring in the 10th week of 2023. (B) The weekly number of deaths attributable to COVID-19 and influenza from the early pandemic to June 2023. During the Omicron-predominant wave, the maximum weekly number of deaths among patients with COVID-19 was 4954, occurring in the 1st week of 2023, whereas the maximum weekly number of deaths among patients with influenza was 192, occurring in the 14th week of 2023. Death was defined as all-cause death within 60 days of a COVID-19 or influenza diagnosis. Wild-type–predominant wave, 1 January 2020–18 April 2021; Alpha-predominant wave, 19 April 2021–18 July 2021; Delta-predominant wave, 19 July 2021–3 January 2022; and Omicron-predominant wave, 4 January 2022–30 June 2023

From January 2020 to June 2023, 29,065,391 patients were diagnosed with COVID-19, and 8,512,666 patients were diagnosed with influenza (Fig. 1A). The weekly number of patients with COVID-19 increased markedly during the Omicron-predominant wave compared to that in the earlier waves. The weekly number of patients with influenza decreased extremely starting in 2020, but the number has rebounded since 2022. The maximum weekly number of patients with COVID-19 in each wave was 32,197 in the wild-type–predominant wave, 29,596 in the Alpha-predominant wave, 113,705 in the Delta-predominant wave, and 1,192,169 in the Omicron-predominant wave. During the Omicron-predominant wave, the maximum weekly number of patients with COVID-19 exceeded the number of patients with influenza (*n* = 486,109).

The weekly numbers of patients with COVID-19 and influenza by age group from January 2020 to June 2023 are presented in Supplementary Fig. 1. During the Omicron-predominant wave, the maximum weekly number of patients with COVID-19 in patients aged 0–19 years (*n* = 306,110) was slightly lower than that of patients with influenza (*n* = 322,215). However, the maximum weekly numbers of patients with COVID-19 in patients aged 20–39, 40–59, 60–79, and ≥ 80 years were larger than the corresponding numbers of patients with influenza.

From January 2020 to June 2023, 134,955 patients with COVID-19 died, versus 9290 patients with influenza (Fig. 1B). The maximum weekly number of deaths among patients with COVID-19 was 769 in the wild-type–predominant wave, 662 in the Alpha-predominant wave, 486 in the Delta-predominant wave, and 4954 in the Omicron-predominant wave. The maximum weekly number of deaths was higher for patients with COVID-19 than for those with influenza (*n* = 192) during the Omicron-predominant wave.

### Patient characteristics and outcomes of patients with COVID-19 by wave

The total number of patients with COVID-19 was 427,387 during the wild-type–predominant wave, 243,797 during the Alpha-predominant wave, 682,597 during the Delta-predominant-wave, and 26,086,367 during the Omicron-predominant wave. The average weekly number of patients with COVID-19 was higher during the Omicron-predominant wave (*n* = 378,848) than during the earlier waves (6312, 18,754, and 28,273 in the wild-type–, Alpha-, and Delta-predominant waves, respectively; Supplementary Fig. 2A). The characteristics and outcomes of patients with COVID-19 by wave are presented in Table 1 and Supplementary Table 2. The proportion of patients receiving respiratory support was lower in the Omicron-predominant wave than in the earlier waves. The mortality rate was lower in the Omicron-predominant wave (0.4%) than in the wild-type (2.7%; risk ratio = 0.16 [95% CI = 0.16–0.16]), Alpha- (2.1%; risk ratio = 0.20 [95% CI = 0.20–0.21]), and Delta-predominant waves (0.7%; risk ratio = 0.62 [95% CI = 0.60–0.63]; Table 1 and Supplementary Fig. 2B). However, the average weekly number of deaths was markedly higher in the Omicron-predominant wave (*n* = 1613) than the earlier waves (169, 397, and 195 in the wild-type–, Alpha-, and Delta-predominant waves, respectively; Supplementary Fig. 2C).

**Table 1:** Patient characteristics and outcomes of COVID–19 by wave

	Wave^a^			
	Wild-type *n* = 427,387	Alpha *n* = 243,797	Delta *n* = 682,597	Omicron *n* = 26,086,367
Age, years	45–49^b^	40–44^b^	30–34^b^	35–39^b^
0–9	26,024 (6.1)	19,389 (8.0)	57,067 (8.4)	3,679,282 (14.1)
10–19	25,812 (6.0)	17,832 (7.3)	76,352 (11.2)	3,603,228 (13.8)
20–29	75,847 (17.7)	45,505 (18.7)	161,436 (23.7)	3,574,122 (13.7)
30–39	58,056 (13.6)	34,764 (14.3)	116,062 (17.0)	3,787,443 (14.5)
40–49	57,366 (13.4)	35,157 (14.4)	110,665 (16.2)	3,912,679 (15.0)
50–59	54,687 (12.8)	31,561 (12.9)	82,918 (12.1)	2,875,429 (11.0)
60–69	40,720 (9.5)	21,366 (8.8)	32,664 (4.8)	1,798,367 (6.9)
70–79	43,286 (10.1)	19,791 (8.1)	23,096 (3.4)	1,425,566 (5.5)
≥ 80	45,589 (10.7)	18,432 (7.6)	22,337 (3.3)	1,430,251 (5.5)
Sex				
Male	228,353 (53.4)	131,697 (54.0)	373,062 (54.7)	12,699,965 (48.7)
Female	199,034 (46.6)	112,100 (46.0)	309,535 (45.3)	13,386,402 (51.3)
Charlson comorbidity index^c^				
0	279,512 (65.4)	170,242 (69.8)	540,711 (79.2)	19,641,911 (75.3)
1–2	105,648 (24.7)	55,400 (22.7)	116,010 (17.0)	5,299,212 (20.3)
3–4	32,501 (7.6)	14,149 (5.8)	20,429 (3.0)	930,034 (3.6)
≥ 5	9726 (2.3)	4006 (1.6)	5447 (0.8)	215,210 (0.8)
Outcome				
Respiratory support care				
Oxygen supplementation	44,923 (10.5)	29,586 (12.1)	45,404 (6.7)	271,992 (1.0)
High-flow nasal cannula	3261 (0.76)	3891 (1.6)	6702 (0.98)	10,888 (0.04)
Mechanical ventilation	6705 (1.6)	3481 (1.4)	4600 (0.67)	16,760 (0.06)
ECMO	334 (0.08)	165 (0.07)	316 (0.05)	324 (0.001)
Death^d^	11,449 (2.7)	5162 (2.1)	4719 (0.7)	111,064 (0.4)

Data are presented as median age category or number (%)

^a^ Wild-type − predominant wave, 1 January 2020–18 April 2021; Alpha-predominant wave, 19 April 2021–18 July 2021; Delta-predominant wave, 19 July 2021–3 January 2022; and Omicron-predominant wave, 4 January 2022–30 April 2023

^b^ Median age category

^c^ One point is assigned if a patient has a disease that belongs to a certain comorbidity category. The Charlson comorbidity index is the total score for each comorbidity category (ranging from 0 to 15 points). For example, if a patient has cerebrovascular disease and renal disease, the index for that patient is 2

^d^ Death was defined as all-cause death within 60 days of a COVID-19 diagnosis

ECMO, extracorporeal membrane oxygenation

**Fig. 2:**
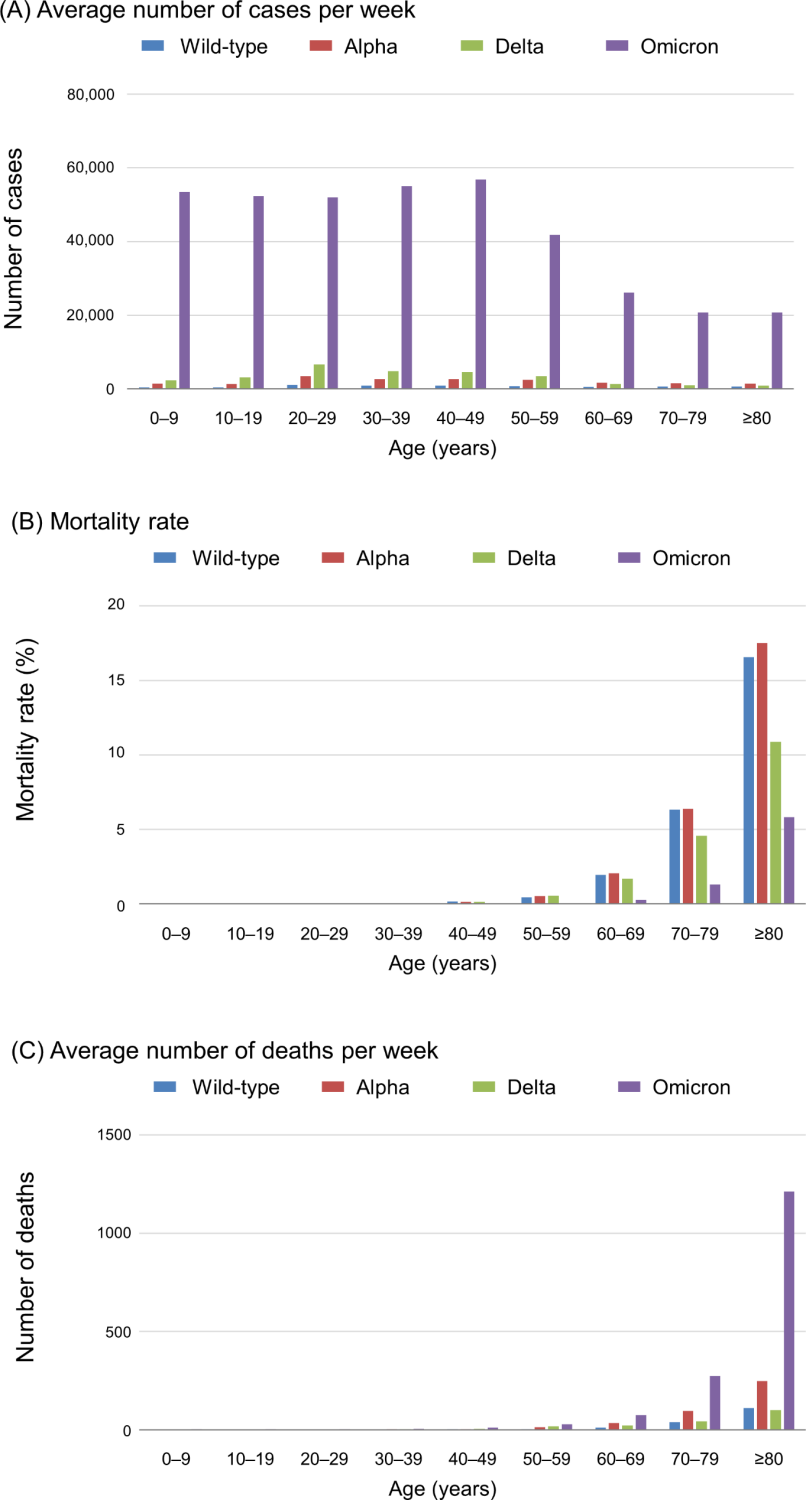
Average number of cases, mortality rates, and average number of deaths among patients with COVID-19 per week by age group and wave. (**A**) The average numbers of patients with COVID-19 per week in the wild-type–, Alpha-, Delta-, and Omicron-predominant waves were 384, 1491, 2364, and 53,434, respectively, among patients aged 0–9 age; 381, 1372, 3163, and 52,329, respectively, among patients aged 10–19; 1120, 3500, 6687, and 51,906, respectively, among patients aged 20–29; 857, 2674, 4807, and 55,004, respectively, among patients aged 30–39; 847, 2704, 4584, and 56,823, respectively, among patients aged 40–49; 808, 2428, 3434, and 41,759, respectively, among patients aged 50–59; 601, 1644, 1353, and 26,117, respectively, among patients aged 60–69; 639, 1522, 957, and 20,703, respectively, among patients aged 70–79; and 673, 1418, 925, and 20,771, respectively, among patients aged ≥ 80. (**B**) The mortality rates in the wild-type–, Alpha-, Delta-, and Omicron-predominant waves were < 0.01%, < 0.01%, < 0.01%, and < 0.01%, respectively, among patients aged 0–19, respectively; <0.1%, < 0.1%, < 0.1%, and < 0.01%, respectively, among patients aged 20–39; 0.16%, 0.14%, 0.14%, and 0.021%, respectively, among patients aged 40–49; 0.45%, 0.53%, 0.55%, and 0.069%, respectively, among patients aged 50–59; 1.95%, 2.07%, 1.71%, and 0.29%, respectively, among patients aged 60–69; 6.32%, 6.39%, 4.58%, and 1.32%, respectively, among patients aged 70–79; and 16.5%, 17.5%, 10.9%, and 5.83%, respectively, among patients aged ≥ 80. (**C**) The average number of deaths of COVID-19 patients per week in the wild-type–, Alpha-, Delta-, and Omicron-predominant waves were < 1, <1, < 1, and 2, respectively, among patients aged 0–9; <1, < 1, <1, and 2, respectively, among patients aged 10–19; <1, < 1, <1, and 3, respectively, among patients aged 20–29; <1, < 1, 2, and 5, respectively, among patients aged 30–39; 1, 4, 6, and 12, respectively, among patients aged 40–49; 4, 13, 19, and 29, respectively, among patients aged 50–59; 12, 34, 23, and 75, respectively, among patients aged 60–69; 40, 97, 44, and 273, respectively, among patients aged 70–79; and 111, 248, 101, and 1212, respectively, among patients aged ≥ 80. Wild-type–predominant wave, 1 January 2020–18 April 2021; Alpha-predominant wave, 19 April 2021–18 July 2021; Delta-predominant wave, 19 July 2021–3 January 2022; and Omicron-predominant wave, 4 January 2022–30 June 2023

In all age groups, the average weekly number of patients with COVID-19 was higher in the Omicron-predominant wave than in the earlier waves (Fig. 2A). The COVID-19 mortality rate increased with age in all waves, but in almost all age groups, the mortality rate was significantly lower in the Omicron-predominant wave than in the earlier waves (Fig. 2B and Supplementary Table 3). The average weekly number of deaths among patients with COVID-19 by age group is presented in Fig. 2C. In patients aged 0–9, 10–19, and 20–29 years, the average number of deaths per week was < 1 in the earlier waves, versus 2–3 in the Omicron-predominant wave. However, in patients aged ≥ 60 years, the number of deaths increased substantially in the Omicron-predominant wave. Specifically, from the Delta-predominant wave to the Omicron-predominant wave, the number of deaths per week increased from 23 to 75 in patients aged 60–69, from 44 to 273 in patients aged 70–79, and from 101 to 1212 in patients aged ≥ 80.

### Characteristics and outcomes of patients with COVID-19 and influenza in the Omicron-predominant wave

Patients with COVID-19 and patients with influenza from 1 May 2022 to 30 April 2023 were compared. During this period, 21,568,390 patients were diagnosed with COVID-19, and 4,508,230 were diagnosed with influenza. To avoid complexity, 137,943 patients who were diagnosed with COVID-19 and influenza on the same day (concurrent infections) were excluded from this analysis. Patient characteristics and outcomes are presented in Table 2 and Supplementary Table 4. The median age category of patients with COVID-19 (35–39 years) was higher than that of patients with influenza (10–14 years; ASD = 0.86). The proportions of patients who received oxygen supplementation, HFNC, and mechanical ventilation were higher in the COVID-19 group than in the influenza group, albeit without significance (ASD < 0.10 for all items).

**Table 2:** Characteristics and outcomes of patients with COVID-19 Omicron and influenza from May 2022 to April 2023

	COVID-19 Omicron *n* = 21,430,447	Influenza *n* = 4,370,287	Absolute standardized difference
Age, years	35–39^a^	10–14^a^	0.86
0–9	2,885,645 (13.5)	1,702,944 (39.0)	
10–19	2,886,194 (13.5)	1,042,471 (23.9)	
20–29	2,852,898 (13.3)	409,937 (9.4)	
30–39	3,061,462 (14.3)	476,589 (10.9)	
40–49	3,211,466 (15.0)	381,687 (8.7)	
50–59	2,457,503 (11.5)	162,634 (3.7)	
60–69	1,570,356 (7.3)	101,011 (2.3)	
70–79	1,255,187 (5.9)	56,339 (1.3)	
≥ 80	1,249,736 (5.8)	36,675 (0.8)	
Sex			0.06
Male	10,388,128 (48.5)	2,255,803 (51.6)	
Female	11,042,319 (51.5)	2,114,484 (48.4)	
Charlson comorbidity index^b^			0.22
0	16,049,783 (74.9)	3,287,038 (75.2)	
1–2	4,398,726 (20.5)	1,033,179 (23.6)	
3–4	798,572 (3.7)	42,456 (1.0)	
≥ 5	183,366 (0.9)	7614 (0.2)	
Outcome			
Respiratory support care			
Oxygen supplementation	213,332 (1.0)	9847 (0.2)	0.09
High-flow nasal cannula	6809 (0.03)	307 (0.01)	0.02
Mechanical ventilation	12,752 (0.06)	823 (0.02)	0.02
ECMO	219 (0.001)	21 (0.001)	< 0.01
Death^c^	90,953 (0.42)	2481 (0.06)	0.08

Data are presented as the median age category or number (%)

^a^ Median age category

^b^ One point is assigned if a patient has a disease that belongs to a certain comorbidity category. The Charlson comorbidity index is the total score for each comorbidity category (ranging from 0 to 15 points)

^c^ Death was defined as all-cause death within 60 days of a COVID-19 or influenza diagnosis

ECMO, extracorporeal membrane oxygenation

**Fig. 3:**
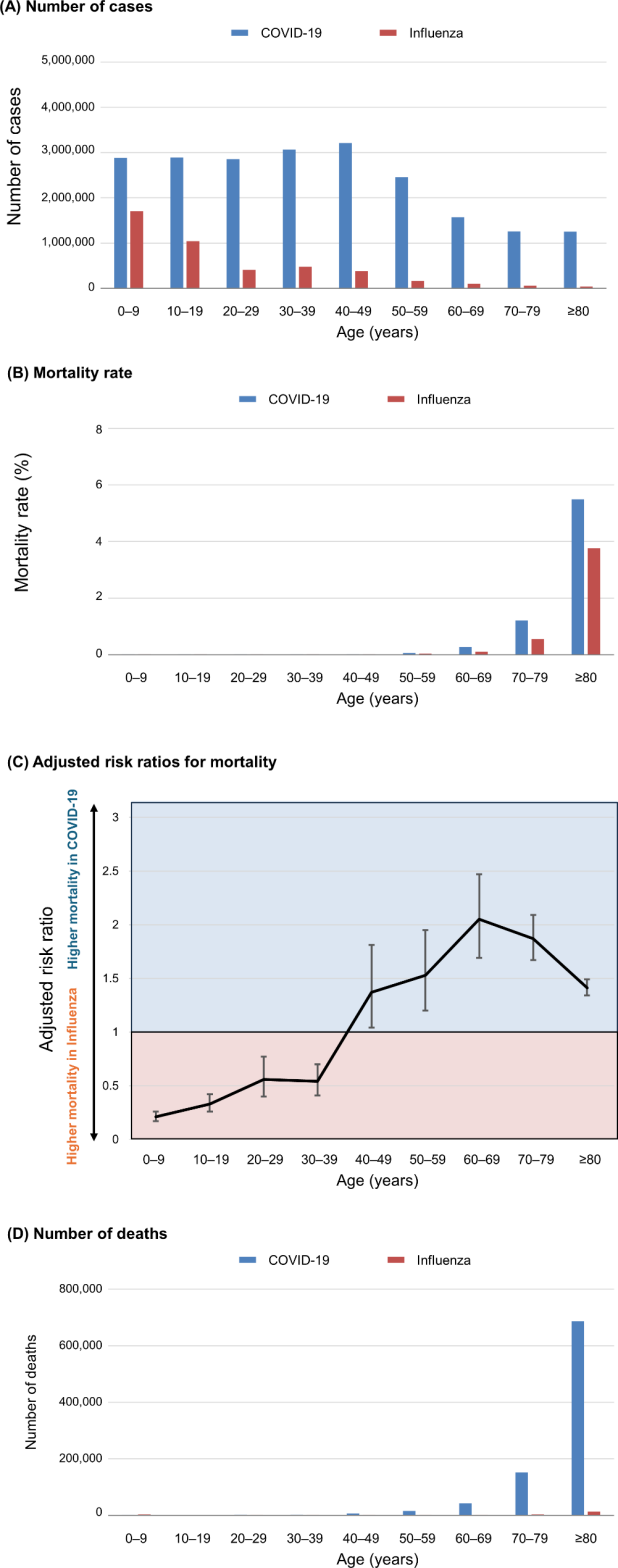
Number of cases, mortality rates, and number of deaths among patients with COVID-19 Omicron and influenza by age group from May 2022 to April 2023. (**A**) Number of cases of COVID-19 Omicron and influenza by age group from May 2022 to April 2023. (**B**) Sixty-day all-cause mortality rates of patients with COVID-19 Omicron and influenza by age group from May 2022 to April 2023. (**C**) Adjusted risk ratios for 60-day all-cause death and the corresponding 95% confidence intervals were plotted. Influenza was used as the reference. The risk ratios were adjusted for sex, cerebrovascular disease, any malignancy, dementia, acquired immunodeficiency syndrome/human immunodeficiency virus, myocardial infarction, renal disease, congestive heart failure, peripheral vascular disease, chronic pulmonary disease, rheumatic disease, peptic ulcer disease, liver disease, diabetes mellitus, hemiplegia or paraplegia, and metastatic solid tumours. (**D**) Number of 60-day all-cause deaths of patients with COVID-19 Omicron and influenza by age group from May 2022 to April 2023

The numbers of patients by age group are presented in Fig. 3A. In all age groups, more patients had COVID-19 than influenza. The 60-day all-cause mortality rates by age group are presented in Fig. 3B. Mortality rates increased with age in both the COVID-19 and influenza groups. The results of the comparison of mortality risk between COVID-19 and influenza are presented in Fig. 3C; Table 3. The adjusted risk ratio of death for COVID-19 against influenza was 0.21 (95% CI = 0.17–0.26) in patients aged 0–9, 0.33 (0.26–0.42) in those aged 10–19, 0.56 (0.40–0.77) in those aged 20–29, 0.54 (0.41–0.70) in those aged 30–39, 1.37 (1.04–1.81) in those aged 40–49, 1.53 (1.20–1.95) in those aged 50–59, 2.05 (1.69–2.47) in those aged 60–69, 1.87 (1.67–2.09) in those aged 70–79, and 1.41 (1.34–1.49) in those aged ≥ 80. The adjusted risk ratio of death for COVID-19 was lower than that for influenza among those aged ≤ 39 years, whereas the risk of death for COVID-19 was higher among those aged ≥ 40 years.

**Table 3:** Mortality rates of COVID-19 Omicron and influenza from May 2022 to April 2023 by age group

	Mortality rate, % (deaths/cases)		Unadjusted risk ratio^a^ (95% CI)	Adjusted risk ratio^a, b^ (95% CI)
	COVID-19 Omicron	Influenza		
Age, years				
0–9	0.004 (109/2,885,645)	0.018 (303/1,702,944)	0.21 (0.17–0.26)	0.21 (0.17–0.26)
10–19	0.004 (117/2,886,194)	0.013 (133/1,042,471)	0.32 (0.25–0.41)	0.33 (0.26–0.42)
20–29	0.006 (179/2,852,898)	0.011 (47/409,937)	0.55 (0.40–0.75)	0.56 (0.40–0.77)
30–39	0.008 (256/3,061,462)	0.015 (73/476,589)	0.55 (0.42–0.71)	0.54 (0.41–0.70)
40–49	0.021 (673/3,211,466)	0.014 (54/381,687)	1.48 (1.12–1.95)	1.37 (1.04–1.81)
50–59	0.064 (1584/2,457,503)	0.042 (68/162,634)	1.54 (1.21–1.96)	1.53 (1.20–1.95)
60–69	0.27 (4219/1,570,356)	0.11 (110/101,011)	2.47 (2.04–2.98)	2.05 (1.69–2.47)
70–79	1.21 (15,189/1,255,187)	0.56 (313/56,339)	2.18(1.95–2.44)	1.87 (1.67–2.09)
≥ 80	5.49 (68,627/1,249,736)	3.76 (1380/36,675)	1.46 (1.39–1.54)	1.41 (1.34–1.49)

^a^ Influenza was used as the reference

^b^ Risk ratios were adjusted for sex, cerebrovascular disease, any malignancy, dementia, acquired immunodeficiency syndrome/human immunodeficiency virus, myocardial infarction, renal disease, congestive heart failure, peripheral vascular disease, chronic pulmonary disease, rheumatic disease, peptic ulcer disease, liver disease, diabetes mellitus, hemiplegia or paraplegia, and metastatic solid tumours

Death was defined as all-cause death within 60 days of a COVID-19 or influenza diagnosis

CI, confidence interval

The number of deaths by age group is presented in Fig. 3D; Table 3. In patients aged ≤ 19 years, the number of deaths was lower for COVID-19 than for influenza. Conversely, the number of deaths was higher for COVID-19 among patients aged ≥ 20 years, and the difference widened considerably in the older groups.

## Discussion

This is the largest study to investigate the number of patients, mortality rates, and deaths during the Omicron-predominant wave of COVID-19. The strengths of this study are that it provided real-world data on patients with COVID-19 since the early pandemic period using the NDB, which contains almost all inpatient and outpatient claims data in Japan, and it compared patients with COVID-19 to those with influenza, the most common epidemic infectious disease, during the Omicron-predominant wave. The mortality rate of COVID-19 decreased significantly over time, but the number of patients was substantially higher in the Omicron-predominant wave than in the earlier waves, resulting in a large increase in the number of deaths, especially among the elderly. In a comparison of patients with COVID-19 and influenza during the Omicron-predominant wave, the mortality rate was lower for patients with COVID-19 than for those with influenza among those aged ≤ 39 years, but the mortality rate was higher for COVID-19 in patients aged ≥ 40 years, especially among the elderly.

This study found that the mortality rate of COVID-19 was lower in the Omicron-predominant wave than in the earlier waves. In previous studies, patients with the COVID-19 Omicron variant had lower mortality rates than those with the Delta variant [3–6]. A population-based study in the United Kingdom (including 440,000 and 1,060,000 patients with COVID-19 Delta and Omicron variants, respectively) reported an approximately 60% reduction in mortality for the Omicron variant compared to the Delta variant [5]. In line with that study, the mortality rate of patients with COVID-19 was 40% lower during the Omicron-predominant wave than during the Delta-predominant wave in this study of approximately 28 million patients. This reduction in mortality was presumably attributable to the decreasing virulence of SARS-Cov-2 virus, as well as the widespread use of vaccines and the development of treatments, including antiviral drugs. However, the average weekly number of deaths attributable to COVID-19 was higher in the Omicron-predominant wave than in the earlier waves. Although the Omicron variant was less virulent than the Delta variant, the former variant displayed increased transmissibility [21]. In a WHO report, the number of patients was higher during the Omicron-predominant wave than during the Delta-predominant wave [22]. In addition, strict infection control measures such as physical distancing and entry restrictions were taken in Japan until the Delta-predominant wave, but these measures were gradually relaxed after this wave. Therefore, we can speculate that the number of patients was markedly higher during the Omicron-predominant wave than during the Delta-predominant wave, resulting in a substantial increase in the number of deaths despite the decreased mortality rate. The observed increases in the number of infected patients and deaths denote an increase in health care resource consumption. Consequently, even in the Omicron-predominant wave, COVID-19 continues to have a significant social impact, and therefore, sustained control measures remain needed against this disease.

In the early stages of the pandemic, older age was reported to be associated with increased mortality in patients with COVID-19 [23–25]. The present study demonstrated that elderly patients with COVID-19 had a higher mortality risk than younger patients, even in the Omicron-predominant wave. An inpatient-based study during the Omicron-predominant wave reported that the mortality rate of elderly patients with COVID-19 was higher than that of elderly patients with influenza [12], in line with the results of the current study including both inpatients and outpatients. The present study found that among the elderly, the number of patients with COVID-19 was considerably higher than the number of those with influenza during the Omicron-predominant wave, resulting in significantly more deaths among elderly patients with COVID-19. As previously noted, the COVID-19 mortality rate decreased during the Omicron-predominant wave, but the total number of deaths increased substantially because of the increased number of infected patients. Notably, the elderly accounted for the majority of this increase in deaths. By contrast, the COVID-19 mortality rate was lower in younger patients despite the higher number of patients. These results suggest that the prevention of COVID-19 (e.g., promotion of vaccination) and aggressive therapeutic interventions remain equally or more important than influenza control in the elderly.

Studies have reported different mortality rates for COVID-19 and influenza depending on age [7, 11, 12]. A study limited to inpatients in the Omicron-predominant wave reported a significantly higher risk of death for COVID-19 than for influenza in patients aged > 65 years, but no significant difference in mortality risk was observed among patients aged ≤ 65 years [12]. The present study included the largest population reported to date, including outpatients and inpatients, and it demonstrated that the risk of death from COVID-19 and influenza differed by age for the first time that in the real world (Fig. 3 and Table 3). Most studies comparing COVID-19 and influenza excluded children from the study population [12–15], and therefore, studies on children have been limited to small numbers of patients [26, 27]. Therefore, the data from the present study, which included children, are significant in this regard. In the present study, the mortality rate and number of deaths attributable to COVID-19 in patients aged 0–19 years were lower than those for their counterparts with influenza during the Omicron-predominant wave. These results suggest that COVID-19 and influenza differ regarding the spread of infection and outcomes between children and adults. Therefore, measures against these infections need to be adjusted by age, and it is possible that measures against influenza should take higher priority than those against COVID-19, especially in children.

This study confirmed a significant decrease in the number of influenza cases in Japan from mid-2020 to late 2022. This period coincided with the COVID-19 pandemic, during which strict public health measures, such as infection prevention protocols and social distancing, were widely implemented. These measures likely played a substantial role in suppressing the spread of influenza during this time. However, in 2023, the number of influenza cases began to rise again. This resurgence may have been influenced by the relaxation of strict public health measures. Nevertheless, the number of influenza cases in 2023 did not reach the levels observed before the COVID-19 pandemic. Several hypotheses may explain this phenomenon. First, the influenza virus strains circulating in 2023 might have been less transmissible than those prevalent before the pandemic. This reduced transmissibility could have contributed to the suppression of case numbers. Second, even after the relaxation of public health measures, preventive behaviors such as mask-wearing and hand hygiene may have been voluntarily maintained by some individuals. These behaviors could have played a role in limiting the spread of influenza to some extent. Lastly, there may have been cross-reactive immunity between COVID-19 and influenza viruses [28–30]. Immune responses induced by SARS-CoV-2 infection or vaccination might have provided partial protection against influenza. Further research is needed to validate these hypotheses.

This study had several limitations. First, the NDB does not record physical findings, blood test or imaging results, or the vaccination status for COVID-19 or influenza. In Japan, following the dominance of the Alpha variant in May 2021, COVID-19 vaccination rates increased, with approximately 40% of the population vaccinated at the peak of the Delta wave and around 80% by the start of the Omicron period [31]. Thus, the decline in COVID-19 mortality observed during the study period may have been influenced not only by viral mutations and advancements in treatment but also by the widespread rollout of vaccination. Second, this study did not include information on the specific causes of death. Therefore, the recorded deaths may have included not only those directly caused by COVID-19 but also those indirectly associated with COVID-19, such as deaths resulting from the exacerbation of comorbidities or other accidental factors, as well as deaths unrelated to COVID-19. Third, the definition of the predominant wave was based on screening data in Tokyo rather than the confirmed variants detected in each patient. Fourth, the association between medications and mortality was outside the scope of this study because the NDB included data on drugs covered by insurance but not those used in clinical trials or specially approved for use without insurance coverage. Finally, asymptomatic or mild cases of COVID-19 may not have sought medical care. As a result, these cases might not have been recorded in the database, potentially leading to an underestimation of the total number of patients and affecting the calculation of mortality rates.

In conclusion, although the mortality rate of COVID-19 was lower during the Omicron-predominant wave than in previous waves, the number of deaths increased substantially because of the higher number of infected patients, especially among the elderly. Although the mortality rate and number of deaths associated with the COVID-19 Omicron variant were lower than those associated with influenza among younger patients, the opposite findings were recorded among elderly patients. Thus, COVID-19 remains associated with increased mortality in the elderly and represents a significant burden to society and healthcare. It is necessary to establish preventive measures and treatments for this disease based on age categories.

## Electronic supplementary material

Below is the link to the electronic supplementary material.

Supplementary Material 1

## Data Availability

No datasets were generated or analysed during the current study.
