# Supplementary material for: Outcomes of COVID-19 in the Omicron-predominant wave: large-scale real-world data analysis with a comparison to influenza
Source: Pneumonia (Nathan). 2025 Feb 5;17:3. doi: 10.1186/s41479-025-00158-y (PMC11796215; doi:10.1186/s41479-025-00158-y)
Supplement: Supplementary file 1 — Supplementary Material 1 [file 41479_2025_158_MOESM1_ESM.docx]

**Supplementary files**

**Supplementary Figure 1. Weekly number of cases of COVID-19 and influenza by age group from the early pandemic to June 2023.**

**Supplementary Figure 2. Average number of cases, mortality rates, and number of deaths for COVID-19 per week by wave.**

**Supplementary Table 1. List of ICD-10 codes for comorbidities listed in the Charlson comorbidity index.**

**Supplementary Table 2. Comorbidities of patients with COVID-19 by wave.**

**Supplementary Table 3. Mortality rates of patients with COVID−19 by wave.**

**Supplementary Table 4. Comorbidities of patients with COVID-19 Omicron and influenza from May 2022 to April 2023.**

**Supplementary reference**

**Supplementary Figure 1. Weekly number of cases of COVID-19 and influenza by age group from the early pandemic to June 2023.**


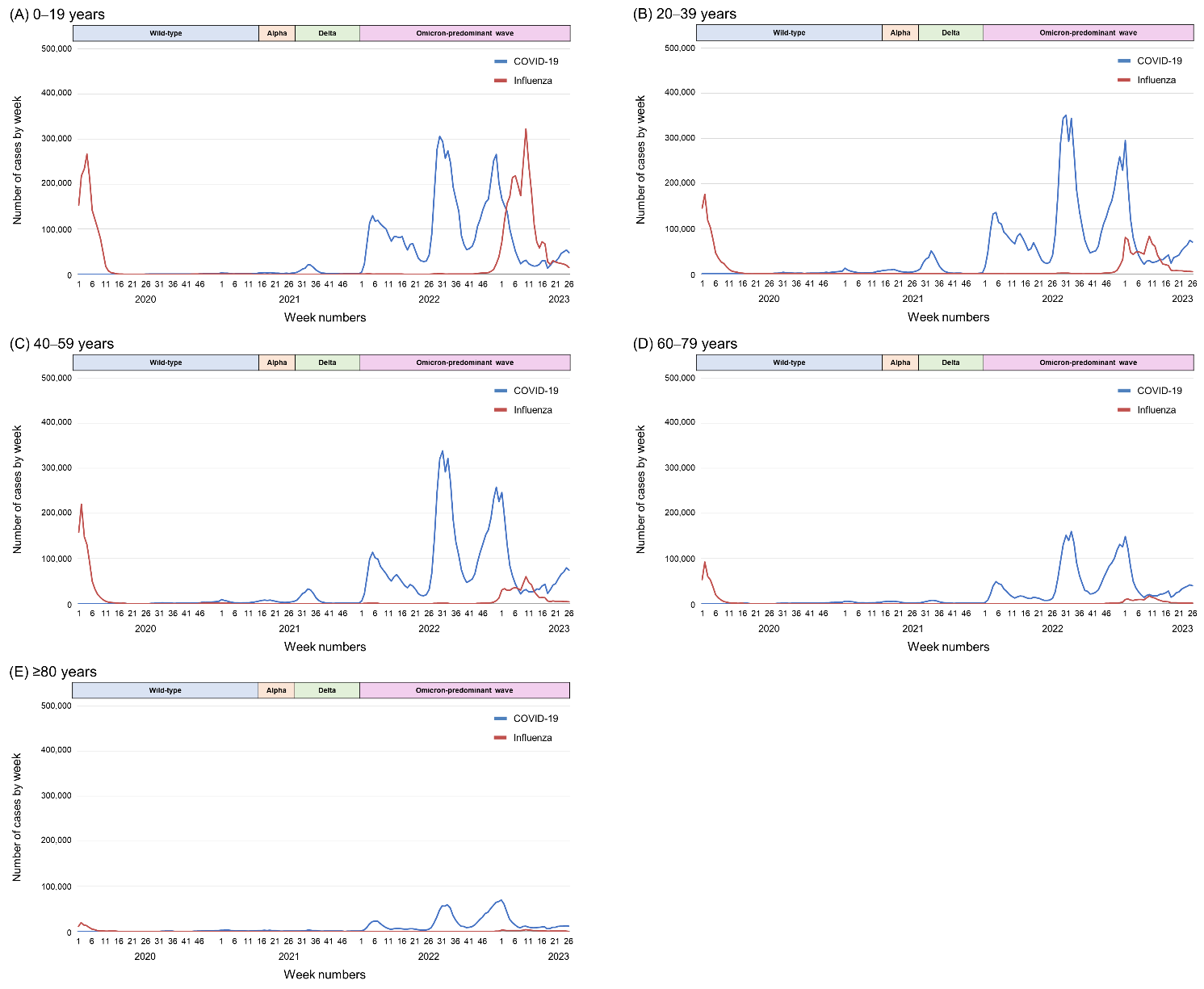


(A) Among patients aged 0–19 years during the Omicron-predominant wave, the maximum weekly number of patients with COVID-19 was 306,110, occurring in the 30th week of 2022, whereas the maximum weekly number of patients with influenza was 322,215, occurring in the 10th week of 2023. (B) Among patients aged 20–39 years during the Omicron-predominant wave, the maximum weekly number of patients with COVID-19 was 351,540, occurring in the 31st week of 2022, whereas the maximum weekly number of patients with influenza was 83,494, occurring in the 10th week of 2023. (C) Among patients aged 40–59 years during the Omicron-predominant wave, the maximum weekly number of patients with COVID-19 was 338,324, occurring in the 31st week of 2022, whereas the maximum weekly number of patients with influenza was 59,964, occurring in the 10th week of 2023. (D) Among patients aged 60–79 during the Omicron-predominant wave, the maximum weekly number of patients with COVID-19 was 160,007, occurring in the 33rd week of 2022, whereas the maximum weekly number of patients with influenza was 16,778, occurring in the 10th week of 2023. (E) Among patients aged ≥80 years during the Omicron-predominant wave, the maximum weekly number of patients with COVID-19 was 69,770, occurring in the 1st week of 2023, whereas the maximum weekly number of patients with influenza was 3658, occurring in the 10th week of 2023.

Wild-type–predominant wave, 1 January 2020–18 April 2021; Alpha-predominant wave, 19 April 2021–18 July 2021; Delta-predominant wave, 19 July 2021–3 January 2022; and Omicron-predominant wave, 4 January 2022–30 June 2023.

**Supplementary Figure 2. Average number of cases, mortality rates, and number of deaths among patients with COVID-19 patients per week by wave.**

**
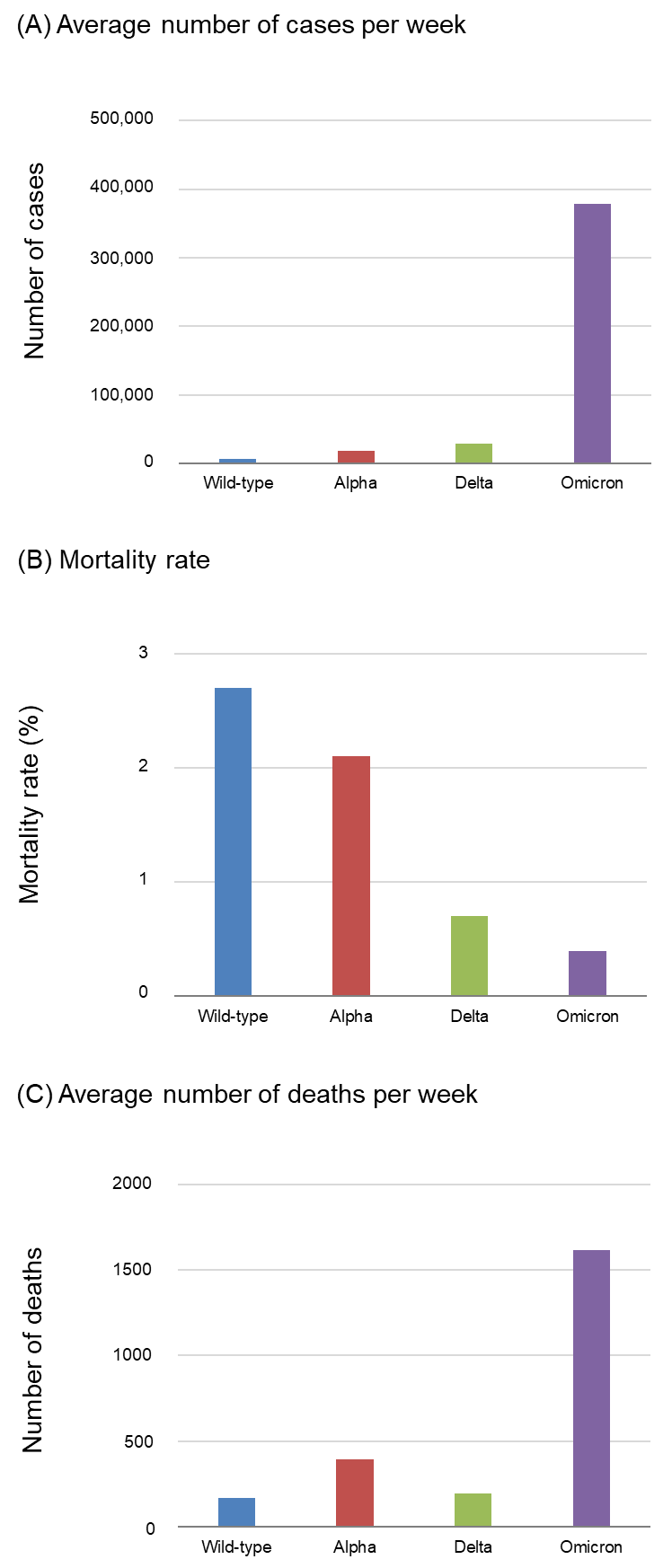
**

(A) During the wild-type–, Alpha-, Delta-, and Omicron-predominant waves, the average weekly numbers of patients with COVID-19 were 6312, 18,754, 28,273, and 378,848, respectively. (B) During the wild-type–, Alpha-, Delta-, and Omicron-predominant waves, the weekly 60-day all-cause mortality rates were 2.7%, 2.1%, 0.7%, and 0.4%, respectively. (C) During the wild-type–, Alpha-, Delta-, and Omicron-predominant waves, the weekly average number of 60-day all-cause deaths were 169, 397, 195, and 1613, respectively.

Wild-type–predominant wave, 1 January 2020–18 April 2021; Alpha-predominant wave, 19 April 2021–18 July 2021; Delta-predominant wave, 19 July 2021–3 January 2022; and Omicron-predominant wave, 4 January 2022–30 June 2023.

**Supplementary Table 1. List of ICD-10 codes for comorbidities listed in the Charlson comorbidity index.**

| Comorbidity | ICD-10 code |
| --- | --- |
| Cerebrovascular disease | G45–46, I60–69, H340 |
| Any malignancy ^a^ | C00–26, C30–34, C37–41, C43, C45–58, C60–76, C81–85, C88, C90–97 |
| Dementia | F00–03, F51, G30, G311 |
| AIDS/HIV | B20–22, B24 |
| Myocardial infarction | I21–22, I252 |
| Renal disease | N18–19, I120, I131, N032–N037, N052–N057, N250, Z490–492, Z940, Z992 |
| Congestive heart failure | I43, I50, I099, I110, I130, I132, I255, I420, I425–429, P290 |
| Peripheral vascular disease | I70–71, I731, I738–739, I771, I790, I792, K551, K558–559, Z958–959 |
| Chronic pulmonary disease | J40–47, J60–67, I278–279, J684, J701, J703 |
| Rheumatic disease | M05–06, M32–34, M315, M 351, M353, M360 |
| Peptic ulcer disease | K25–28 |
| Liver disease | B18, I850, I859, I864, I982, K700–704, K709, K711, K713–715, K717, K721, K729, K760, K762–769, Z944 |
| Diabetes mellitus | E10–14 |
| Hemiplegia or paraplegia | G81–82, G041, G144, G801–802, G830–834, G839 |
| Metastatic solid tumours | C77–80 |

^a^ Any malignancy, including lymphoma and leukemia but excluding malignant neoplasm of the skin

Coding algorithms for the Charlson comorbidity index were cited from Quan et al [1].

AIDS, acquired immunodeficiency syndrome; HIV, human immunodeficiency virus

1 Quan H, Sundararajan V, Halfon P, et al. Coding algorithms for defining comorbidities in ICD-9-CM and ICD-10 administrative data. Med Care 2005; 43(11): 1130-9.

**Supplementary Table 2. Comorbidities of patients with COVID-19 by wave.**

|  | **Wave^a^** | | | |
| --- | --- | --- | --- | --- |
|  | **Wild-type**  **n =** **427,387** | **Alpha**  **n =** **243,797** | **Delta**  **n =** **682,597** | **Omicron**  **n =** **26,086,367** |
| Charlson comorbidity |  |  |  |  |
| Cerebrovascular disease | 30,244 (7.1) | 13,072 (5.4) | 17,793 (2.6) | 950,979 (3.6) |
| Any malignancy | 23,049 (5.4) | 10,131 (4.2) | 16,279 (2.4) | 703,558 (2.7) |
| Dementia | 17,943 (4.2) | 7106 (2.9) | 7980 (1.2) | 589,207 (2.3) |
| AIDS/HIV | 212 (0.05) | 76 (0.03) | 196 (0.03) | 3162 (0.01) |
| Myocardial infarction | 4891 (1.1) | 2194 (0.9) | 3132 (0.5) | 128,865 (0.5) |
| Renal disease | 11,687 (2.7) | 5151 (2.1) | 7481 (1.1) | 353,905 (1.4) |
| Congestive heart failure | 32,052 (7.5) | 13,990 (5.7) | 20,848 (3.1) | 1,002,065 (3.8) |
| Peripheral vascular disease | 6276 (1.5) | 2935 (1.2) | 4661 (0.7) | 201,005 (0.8) |
| Chronic pulmonary disease | 59,411 (13.9) | 31,129 (12.8) | 68,995 (10.1) | 3,065,356 (11.8) |
| Rheumatic disease | 6740 (1.6) | 3216 (1.3) | 5754 (0.8) | 254,013 (1.0) |
| Peptic ulcer disease | 30,447 (7.1) | 13,814 (5.7) | 24,417 (3.6) | 923,929 (3.5) |
| Liver disease | 33,719 (7.9) | 16,744 (6.9) | 29,603 (4.3) | 1,199,928 (4.6) |
| Diabetes mellitus | 44,145 (10.3) | 20,834 (8.5) | 32,609 (4.8) | 1,359,226 (5.2) |
| Hemiplegia or paraplegia | 2170 (0.5) | 895 (0.4) | 1337 (0.2) | 66,861 (0.3) |
| Metastatic solid tumours | 3950 (0.9) | 1669 (0.7) | 2943 (0.4) | 95,603 (0.4) |

Data are presented as number (%).

^a^ Wild-type–predominant wave, 1 January 2020–18 April 2021; Alpha-predominant wave, 19 April 2021–18 July 2021; Delta-predominant wave, 19 July 2021–3 January 2022; and Omicron-predominant wave, 4 January 2022–30 June 2023.

AIDS, acquired immunodeficiency syndrome; HIV, human immunodeficiency virus

**Supplementary Table 3. Mortality rates of patients with COVID−19 by wave.**

|  | **Wave^a^** | | | |  | **Omicron vs. Wild-type^b^** | |  | **Omicron vs. Alpha^b^** | |  | **Omicron vs. Delta^b^** | |  |
| --- | --- | --- | --- | --- | --- | --- | --- | --- | --- | --- | --- | --- | --- | --- |
|  | Wild-type,  Mortality rate, %  (Deaths/cases^c^) | Alpha,  Mortality rate, %  (Deaths/cases^c^) | Delta,  Mortality rate, %  (Deaths/cases^c^) | Omicron,  Mortality rate, %  (Deaths/cases^c^) |  | Unadjusted  risk ratio  (95% CI) | Adjusted  risk ratio^d^  (95% CI) |  | Unadjusted  risk ratio  (95% CI) | Adjusted  risk ratio^d^  (95% CI) |  | Unadjusted  risk ratio  (95% CI) | Adjusted  risk ratio^d^  (95% CI) |  |
| Age, years |  |  |  |  |  |  |  |  |  |  |  |  |  |  |
| 0–19^e^ | <0.01  (<1/765) | <0.01  (<1/2863) | <0.01  (<1/5527) | <0.01  (4/105,763) |  | NS | 0.44  (0.18–1.07) |  | NS | 0.53  (0.17–1.66) |  | NS | 0.63  (0.31–1.27) |  |
| 20–39^e^ | <0.1  (<1/1977) | <0.1  (<1/6174) | <0.1  (2/11,494) | <0.01  (8/106,910) |  | NS | 0.29  (0.21–0.41) |  | NS | 0.58  (0.32–1.05) |  | NS | 0.35  (0.27–0.46) |  |
| 40–49 | 0.16  (1/ 847) | 0.14  (4/2704) | 0.14  (6/4584) | <0.1  (12/56,823) |  | 0.13  (0.11–0.17) | 0.20  (0.16–0.25) |  | 0.15  (0.12–0.21) | 0.21  (0.16–0.28) |  | 0.15  (0.13–0.18) | 0.18  (0.15–0.21) |  |
| 50–59 | 0.45  (4/808) | 0.53  (13/2428) | 0.55  (19/3434) | <0.1  (29/41,759) |  | 0.15  (0.13–0.18) | 0.24  (0.21–0.28) |  | 0.13  (0.11–0.15) | 0.18  (0.16–0.21) |  | 0.13  (0.11–0.14) | 0.15  (0.13–0.16) |  |
| 60–69 | 1.95  (12/601) | 2.07  (34/1644) | 1.71  (23/1353) | 0.29  (75/26,117) |  | 0.15  (0.14–0.16) | 0.23  (0.21–0.25) |  | 0.14  (0.13–0.15) | 0.18  (0.16–0.20) |  | 0.17  (0.15–0.18) | 0.22  (0.20–0.24) |  |
| 70–79 | 6.32  (40/639) | 6.39  (97/1522) | 4.58  (44/957) | 1.32  (273/20,703) |  | 0.21  (0.20–0.22) | 0.25  (0.24–0.26) |  | 0.21  (0.20–0.22) | 0.23  (0.22–0.24) |  | 0.29  (0.27–0.31) | 0.35  (0.33–0.37) |  |
| ≥80 | 16.5  (111/673) | 17.5  (248/1418) | 10.9  (101/925) | 5.83  (1212/20,771) |  | 0.35  (0.34–0.36) | 0.36  (0.35–0.37) |  | 0.33  (0.32–0.34) | 0.33  (0.32–0.35) |  | 0.54  (0.52–0.56) | 0.54  (0.52–0.56) |  |

Death was defined as all-cause death occurring within 60 days of a COVID-19 diagnosis.

^a^ Wild-type–predominant wave, 1 January 2020–18 April 2021; Alpha-predominant wave, 19 April 2021–18 July 2021; Delta-predominant wave, 19 July 2021–3 January 2022; and Omicron-predominant wave, 4 January 2022–30 June 2023.

^b^ The earlier wave was used as the reference.

^c^ Average number of cases and deaths among patients with COVID-19 per week.

^d^ Risk ratios were adjusted for sex, cerebrovascular disease, any malignancy, dementia, acquired immunodeficiency syndrome/human immunodeficiency virus, myocardial infarction, renal disease, congestive heart failure, peripheral vascular disease, chronic pulmonary disease, rheumatic disease, peptic ulcer disease, liver disease, diabetes mellitus, hemiplegia or paraplegia, and metastatic solid tumours.

^e^ JMHLW guidelines for the use of claims data require that variables with fewer than 10 cases must not be published. Because the 0–9, 10–19, 20–29, and 30–39 categories contained fewer than 10 cases, they were combined into the 0–19 and 20–39 categories, and the corresponding unadjusted risk ratios were not presented.

JMHLW, Japan Ministry of Health, Labor, and Welfare; NS, not shown.

**Supplementary Table 4. Comorbidities of patients with COVID-19 Omicron and influenza from May 2022 to April 2023.**

|  | **COVID-19 Omicron**  **n = 21,430,447** | **Influenza**  **n =** **4,370,287** | **Absolute standardized difference** |
| --- | --- | --- | --- |
| Comorbidity |  |  |  |
| Cerebrovascular disease | 820,899 (3.8) | 35,118 (0.8) | 0.20 |
| Any malignancy | 606,799 (2.8) | 34,506 (0.8) | 0.15 |
| Dementia | 506,885 (2.4) | 12,524 (0.3) | 0.18 |
| AIDS/HIV | 2572 (0.01) | 304 (0.01) | 0.01 |
| Myocardial infarction | 110,174 (0.5) | 4811 (0.1) | 0.07 |
| Renal disease | 305,119 (1.4) | 14,908 (0.3) | 0.12 |
| Congestive heart failure | 862,759 (4.0) | 45,887 (1.1) | 0.19 |
| Peripheral vascular disease | 170,982 (0.8) | 12,049 (0.3) | 0.07 |
| Chronic pulmonary disease | 2,477,849 (11.6) | 900,260 (20.6) | 0.25 |
| Rheumatic disease | 218,601 (1.0) | 15,266 (0.4) | 0.08 |
| Peptic ulcer disease | 780,792 (3.6) | 62,280 (1.4) | 0.14 |
| Liver disease | 1,024,089 (4.8) | 82,221 (1.9) | 0.16 |
| Diabetes mellitus | 1,168,380 (5.5) | 70,318 (1.6) | 0.21 |
| Hemiplegia or paraplegia | 56,604 (0.3) | 3,250 (0.07) | 0.05 |
| Metastatic solid tumours | 81,657 (0.4) | 4446 (0.1) | 0.06 |

Data are presented as number (%).

AIDS, acquired immunodeficiency syndrome; HIV, human immunodeficiency virus

**Supplementary reference**

1 Quan H, Sundararajan V, Halfon P, et al. Coding algorithms for defining comorbidities in ICD-9-CM and ICD-10 administrative data. Med Care. 2005; 43(11): 1130-9.
